# Evolving concepts in HER2-low breast cancer: Genomic insights, definitions, and treatment paradigms

**DOI:** 10.18632/oncotarget.28680

**Published:** 2025-01-20

**Authors:** Whitney L. Hensing, Emily L. Podany, James J. Sears, Shaili Tapiavala, Andrew A. Davis

**Affiliations:** ^1^Saint Luke’s Cancer Institute, University of Missouri-KC School of Medicine, Kansas City, MO 64111, USA; ^2^Department of Medicine, Division of Oncology, Washington University in St. Louis School of Medicine, St. Louis, MO 63110, USA

**Keywords:** breast cancer, HER2-low, genomics

## Abstract

Human epidermal growth factor receptor 2 (HER2)-low breast cancer has recently emerged as a new clinical subtype of breast cancer, benefiting from treatment with novel anti-HER2 antibody-drug conjugates. Emergence of HER2-low as a clinical subtype has raised several important questions related to the (i) biology and prognostic significance of HER2-low breast cancer, (ii) definition of HER2-low breast cancer, and (iii) clinical management of HER2-low breast cancer within existing treatment paradigms. Previously, our group evaluated the genomic landscape and prognostic significance of HER2-low using circulating tumor DNA in patients with metastatic breast cancer, determining that HER2-low did not represent a unique biologic subtype. In this review, we discuss our findings in the context of existing literature on HER2-low breast cancer and propose how best to translate current evidence into clinical management of HER2-low breast cancer.

## INTRODUCTION

At least half of all breast cancers harbor low HER2 expression, which can be targeted with the anti-HER2 antibody-drug conjugate (ADC) trastuzumab deruxtecan (T-DXd), leading to significant survival benefit in the metastatic setting [1]. This has led to a paradigm shift in breast cancer, which had been historically classified as HER2-negative or HER2-positive. An update to the American Society of Clinical Oncology/College of American Pathologists (ASCO/CAP) guidelines for HER2 testing was published in 2023 including a definition for “HER2-low” [2, 3]. “HER2-low” is defined as tumors without HER2 overexpression, but with immunohistochemistry (IHC) 1+ or 2+ and without amplification by in-situ hybridization (ISH) [3]. Among the IHC 0 category, tumors may exhibit a complete lack of expression or faint to barely perceptible incomplete membrane staining in up to 10% of tumor cells (“HER2-ultralow”). Clinical relevance of the HER2 “ultra-low” category has emerged recently with publication of DESTINEY-Breast06 [4]; however, this category of HER2 expression is not included in the existing guidelines.

Several challenges remain in the interpretation of HER2-low breast cancer, including the biological impact of low HER2 expression, the definition of HER2-low, and how best to integrate anti-HER2 ADCs into existing treatment algorithms. In a recent study published by our group in Clinical Cancer Research [5], we evaluated the landscape of circulating tumor DNA (ctDNA) genetic alterations and prognosis across the spectrum of HER2 expression in patients with metastatic breast cancer (MBC) to determine if HER2-low represented a distinct biologic subtype. In this review, we discuss our findings in the context of current knowledge regarding HER2-low breast cancer and the evolving definition of HER2-low. We also propose how best to translate existing data on HER2-low breast cancer into the clinical setting.

## BIOLOGICAL AND PROGNOSTIC SIGNIFICANCE OF LOW HER2 EXPRESSION: IS HER2-LOW A UNIQUE SUBTYPE?

While the biological and prognostic impact of HER2 overexpression in HER2-positive breast cancer is well-known, the impact of low HER2 expression has not been well-defined. Several retrospective studies, predominantly tissue based, have evaluated the genomics of HER2-low versus HER2-0 tumors (Table 1) to define whether HER2-low is a unique biological and clinical subtype. Other studies have assessed the prognostic impact of low HER2 expression (Table 2). Findings recently published by our group [5], as well as results from the existing literature, have helped shed light on this question and, in our view, confirm that HER2-low breast cancer is unlikely to represent a unique biologic or clinical subtype but rather a therapeutic target for a new class of HER2 targeted therapy: HER2-directed ADCs.

**Table 1:** Summary of genomic findings in HER2-low breast cancer from existing literature, including key genomic differences between HER2-low and HER2-0 tumors

References	Size of cohort	Key molecular differences between HER2-low and HER2-0 tumors	Most frequent genomic alterations in HER2-low tumors	Key genomic differences between HER2-low and HER2-0 tumors	Key genomic differences stratified by HR status
Agostinetto et al., 2021 [15]	804 pts with BC	HER2-low: 81.9% HR+ HR+: 65.9% LumA HR−: 76.7% Basal-like HER2-0: 75.5% HR+ HR+: 69.9% LumA HR−: 90.6% Basal-like	Not reported	Significantly higher *ERBB2* expression in HER2-low No differences in *ESR1* expression	Significantly higher *ERBB2* expression in HR+/HER2-low vs. HR−/HER2-low *ESR1* expression higher in all HR+ subtypes
Denkert et al., 2021 [8]	2310 pts with HER2- negative BC	HER2-low: 64.0% HR+ HER2-0: 36.7% HR+	*TP53* (33.4%) *PIK3CA* (24.8%)	Significantly higher rates of *PIK3CA* mutations in HER2 low Significantly lower rates of *TP53* mutations in HER2-low	HR+: significantly higher rates of *TP53* mutations in HER2-0, no difference in *PIK3CA* mutations HR−: no significant difference in *TP53* or *PIK3CA* mutations
Schettini et al., 2021 [16]	3689 pts with HER2- negative BC	HER2-low: 88.2% HR+ 50.8% LumA 28.8% LumB 13.3% Basal-like HER2-0: 69.6% HR+ 28.7% LumA 18.9% LumB 43.7% Basal-like	Not reported	Higher expression in HER2-low: *ESR1, ERBB2, BCL2* Lower expression in HER2-low: *CCNE1*	HR+: significantly higher *ERBB2* expression in HER2-low HR−: no significant genetic differences
Berrino et al., 2022 [21]	99 pts with HER2-low BC	HER2-low: 88.9% ER+ 29.3% LumA 50.5% LumB 6.1% Basal-like	*PIK3CA* (33%) *GATA3* (18%) *TP53* (17%) *ERBB*2 (8%)	Higher frequency of *SPN* mutations in HER2-low Lower frequency of *ESR1* mutations in HER2-low	Not reported
Zhang et al., 2022 [22]	281 pts with BC	HER2-low: 93.1% ER+ ER+: 70.4% LumA ER-: 66.7% Basal-like HER2-0: 82.9% ER+ ER+: 75.7% LumA ER-: 89.3% Basal-like	Not reported	Not reported	Not reported
Hensing et al., 2023 [2]	749 pts with mBC	HER2-low: 85.3% HR+ HER2-0: 81.3% HR+	*TP53* *PIK3CA* (33%) *ESR1*	Significantly higher rates of *PIK3CA* mutations in HER2-low No difference in oncogenic pathways	HR+: *PIK3CA* mutations more common in HER2-low HR−: not analyzed due to small number
Jin et al., 2023 [17]	579 pts with mBC	HER2-low: 63.7% HR+ HER2-0: 42.2% HR+	*TP53* (62%) *PIK3CA* (45%) *CCND1, FGF3, KMT2C*, (17%) *FGF19* (16%) *ESR1, FGF4* (15%) *GATA3* (13%)	*TP53* mutations less common in HER2-low	HR+: *SETD2, ESR1, ARID1A* mutations less common in HER2-low HR−: *SLX* mutations more common in HER2-low Among HER2-low, *TP53* mutations enriched in HR− and *ESR1*, *ERBB2* enriched in HR+
Dai et al., 2023 [9]	434 pts with HER2-low BC	HER2-low: 83.2% HR+ 13.8% Basal-like HR−: 69.7% Basal-like HER2-0: 69.2% HR+ 35.2% Basal-like HR−: 96.3% Basal-like	*PIK3CA* (43%) *TP53* (33%) *GATA3* (14%) *MAP3K1* (12%)	Not reported	HR−: HER2-low non-basal-like had more *PIK3CA* mutations (61.5%) than HER2-low basal-like (10.8%) and HER2-0 (9.1%) HR+: Frequency of mutations (HER2-low, HER2-0): □ *PIK3CA*: 45%, 39% □ *TP53:* 24%, 36% □ *GATA3:* 16%, 9% □ *MAP3K1*: 13%, 7%
Tarantino P et al., 2023 [20]	1039 pts with mBC	HER2-low: 80.5% HR+ HER2-0: 66.9% HR+	*TP53* (79.8%) *PIK3CA* (32.9%) *CDH1* (15.3%) *GATA3* (13.5%) *ESR1* (10.8%)	Higher number of *ERBB2* alleles in HER2-low Higher number of *ERBB2* hemi-deletions in HER2-0 No significant difference in distribution of common mutations in overall genomic profiles *MTOR* mutations, *ESR1* and *IGF1R* amplifications enriched in HER2-low *MAP3K1, NF1,* and *TP53* mutations enriched in HER2-0	Only marginal differences in genomic landscape after correcting for ER expression
Tsai et al., 2024 [7]	615 pts with HER2- negative BC	HER2-low: 87.5% ER+ HER2-0: 70.3% ER+	*PIK3CA* (17.62%)	Higher frequency of *PIK3CA* single nucleotide alteration in HER2-low Lower frequency of copy number alterations in *PIK3CA*, *CCND3*, and *CCND2* in HER2-low	Not reported

Abbreviations: HR: hormone receptor; BC: breast cancer; mBC: metastatic breast cancer; LumA: Luminal A; LumB: Luminal B; ER: estrogen receptor.

**Table 2:** Summary of prognostic findings in HER2-low breast cancer from existing literature, including comparison of outcomes between HER2-low and HER2-0

References	Size of Cohort	Prognostic findings in HER2-low vs. HER2-0	Prognostic findings stratified by HR status
Agostinetto et al., 2021 [15]	804 pts with BC	No significant differences in DFS, PFS, or OS	Pts with HR− tumors had significantly worse PFS vs. HR+ in both HER2-low and HER2-0
Denkert et al., 2021 [8]	2310 pts with HER2-non-amplified BC	Longer DFS and OS in HER2-low	HR+: Lower pCR in HER2-low tumors vs. HER2-0; No survival difference by HER2 status HR−: No pCR difference; HER2-low significantly longer DFS and OS
Hein et al., 2021 [34]	3159 pts with BC	No difference in PFS or OS	Not reported
Jacot et al., 2021 [28]	296 pts with BC	No significant differences in OS and RFS	Not reported
Mutai et al., 2021 [30]	608 pts with BC	Pts with high genomic risk (RS >25) had significantly better OS and DFS if HER2-low Lower genomic risk (RS ≤25) had no significant association	Not reported
Schettini et al., 2021 [16]	1304 pts with HER2-negative BC	No significant differences in OS	No significant differences in OS when stratified by HR status
Won et al., 2022 [23]	30,491 pts with BC	HER2-low had significantly better BCSS No significant difference in OS	Patients with HR− tumors had worse OS and BCSS than those with HR+
Tan et al., 2022 [24]	28,280 pts with BC	HER2-low had significantly better OS and RFS HER2 IHC 1+ had better RFS and OS vs. HER2-0, however HER2 IHC 2+ did not	HR+: Pts with HER2-low tumors had better RFS and OS vs. HER2-0 HR−: Pts with HER2-low tumors had better OS vs. HER2-0 but no difference in RFS
Horisawa et al., 2022 [25]	4918 pts with BC	No significant difference in DFS or OS	No significant difference in DFS or OS when stratified by HR-status
Li et al., 2022 [32]	1433 pts with BC	Longer OS for pts with HER2-low tumors vs. HER2-0	HR+: Longer OS for pts with HER2-low tumors vs. HER2-0 HR−: No significant difference in OS
Domergue et al., 2022 [27]	437 pts with BC	No difference in pCR, OS, or DFS between HER2-low and HER2-0	Not reported
de Calbiac et al., 2022 [31]	15,054 pts with mBC	Longer OS for pts with HER2-low tumors No difference in PFS	No significant difference in OS when stratified by HR status
Almstedt et al., 2022 [36]	410 pts with BC	HER2-low pts had significantly longer OS and DFS	HR+: HER2-low pts had significantly longer OS and DFS vs. HER2-0 HR−: No difference in OS, significantly longer DFS
Jin et al., 2023 [17]	579 pts with mBC	Pts with HER2-low tumors had longer DFS and OS	No significant difference in OS or DFS when stratified by HR status
Dai et al., 2023 [9]	434 pts with HER2-low BC	Higher DMFS in HER2-low Compared with HER2-0 No significant difference in OS	HR+: HER2-low had higher DMFS vs. HER2-0 HR−: No difference in DMFS
Baez-Navarro et al., 2024 [37]	11,988 pts with BC	No significant difference in OS	No significant difference in OS when stratified by HR status

Abbreviations: HR: hormone receptor; BC: breast cancer; mBC: metastatic breast cancer; DFS: disease free survival; PFS: progression free survival; OS: overall survival; pCR: pathologic complete response; RFS: recurrence free survival; DMFS: distant metastasis free survival.

## GENOMIC DATA IN HER2-LOW BREAST CANCERS

Utilizing a retrospective cohort of patients with MBC from three academic centers, we compared the landscape of genomic alterations detected by ctDNA across the spectrum of HER2 expression: tumors without HER2 expression (HER2-0), low level HER2 expression (HER2-low) and tumors with HER2 overexpression (HER2-positive). We found an increased prevalence of *PIK3CA* alterations in HER2-low MBC compared to HER2-0 MBC (33% vs. 23%, *p* = 0.024). However, we found no difference in *ERBB2* alterations or oncogenic pathways between HER2-low and HER2-0 MBC. Consistent with other studies, most HER2-low MBCs were also hormone receptor (HR)-positive. Among those with HR-positive MBC, we again found a higher prevalence of *PIK3CA* alterations (35% vs. 26%, *p* = 0.03), but no difference in *ERBB2* alterations, oncogenic pathways, or prognosis between HER2-low and HER2-0 MBC.

*PIK3CA* alterations are common in HR-positive, HER2-negative breast cancers, occurring in up to 30–40% [6], and several studies have found these alterations to be more frequent in HER2-low compared with HER2-0 MBC [5, 7–9] (Table 1). In our retrospective cohort, *PIK3CA* mutations were present in 28% of all samples including 30% of HR-positive and 20% of triple-negative breast cancer (TNBC) subtypes [5]. *PIK3CA* mutations have been associated with poor prognosis in HR-positive breast cancer [10, 11], and have been associated with resistance to HER2-targeted therapies in HER2-positive breast cancer [12, 13] However, the clinical significance of *PIK3CA* mutations in HER2-low breast cancers is unknown. In the T-DXd and control arms of DESTINY-Breast04, 36.1% and 41.6% of patients, respectively, had *PIK3CA* mutations found on baseline ctDNA. T-DXd was superior to treatment of physician’s choice (TPC) regardless of *PIK3CA* mutation status [14].

In our cohort, we did not identify any difference in *ERBB2* alterations between HER2-low and HER2-0 MBC [5]. However, some tissue-based studies have identified higher rates of *ERBB2* alterations among HER2-low compared with HER2-0 breast cancers [15–17]. Interestingly, in these studies the association is found only in the HR-positive subgroup. Cross talk between ER and HER2 pathways could result in modulation of HER2 expression [18, 19]. A retrospective analysis by Schettini et al., including PAM50 genomic data from 3689 patients, found higher expression of *ERBB2* and luminal-related genes among HR-positive HER2-low breast cancers, suggesting a distinct genomic profile. However, no genes were differentially expressed in HR-negative breast cancers according to HER2 status [16]. Other studies have identified associations between HR-positive HER2-low breast cancers and the luminal intrinsic subtype [8, 15] (Table 1). In a Taiwan-based study by Tsai et al., genetic alterations detected by tissue-based next-generation sequencing (NGS) were compared in 615 HER2 negative, predominately early-stage breast cancers. Compared with HER2-0, significantly more HER2-low breast cancers were HR-positive. When authors compared TNBC to HR-positive breast cancers, there was a higher frequency of *ERBB2* alterations in the HR-positive subgroup (17.0% vs. 28.2%; *p* = 0.014) [7]. However, the authors did not compare genomic alterations between HR-positive or TNBC in the HER2-low and HER2-0 samples. Thus, it is unclear how the higher incidence of HR-positive among HER2-low breast cancers influences these results. Table 1 summarizes differences in genomic profiles between HER2-low and HER2-0 from the existing literature, including stratified analyses by HR-status, when available.

Data are limited on the uniqueness of the HR-negative HER2-low subgroup, perhaps due to smaller sample sizes in retrospective studies. In our cohort, we identified only 162 patients with HR-negative MBC, including 52 HER2-low and 42 HER2-0. There were no differences in genetic alterations by ctDNA between HER2-low and HER2-0 in the HR-negative group [5]. Prior studies have shown that HR-negative HER2-low tumors most commonly have basal-like intrinsic molecular subtypes; however, there may be a higher proportion of non-basal-like tumors among HR-negative HER2-low compared to HR-negative HER2-0 (Table 1). In a Chinese cohort study, the proportion of non-basal-like tumors was significantly higher in HR-negative HER2-low compared with HR-negative HER2-0 breast cancers (30.3% vs. 3.7%, *p* = 0.005), and particularly prevalent in the HER2 immunohistochemistry (IHC) 2+ subgroup (57.1%) [9]. Although the non-basal-like tumors mostly consisted of HER2-enriched tumors, there was no difference in *ERBB2* alterations between subtypes [9]. These findings suggest that HR-negative HER2-0 may represent the more typical, basal-like subtype characteristic of TNBC than HR-negative HER2-low tumors, and more studies are needed to confirm these associations.

As demonstrated in Table 1, differences in PAM50 intrinsic subtypes appear to be driven primarily by HR-status rather than level of HER2 expression [16]. Similarly, studies comparing mutational landscapes between HER2-0 and HER2-low breast cancers have shown very few differences when considering HR-status. Tissue-based NGS profiles of 445 MBCs were compared among six subtypes defined by HR and HER2 status. There were significantly less differentially mutated genes between HR-positive HER2-low/HR-positive HER2-0 and HR-negative HER2-low/HR-negative HER2-0, than between other subtypes. When mutational and copy number variation data were combined as a whole profile using non-negative matrix factorization (NMF) analysis, three distinct molecular clusters were identified. There was no difference in the distribution of mutational clusters between HER2-low and HER2-0 breast cancers, while HER2 positive breast cancers demonstrated a predominately cluster 1 phenotype [17]. Our study using ctDNA-based NGS data from 749 patients with MBC revealed similar findings, with very few differences in genetic alterations and oncogenic pathways between HER2-0 and HER2-low breast cancer [5]. Another cohort study, including 1039 patients with HER2 negative MBC from the Dana Farber Cancer Institute, found no significant differences in the distribution of genomic alterations between HER2-low and HER2-0 tumors, after adjusting for multiple hypothesis testing. This was also true when the analysis was stratified by ER-status and when comparing IHC 2+ samples and IHC 0 samples [20].

## PROGNOSTIC IMPACT OF LOW HER2 EXPRESSION IN BREAST CANCER

Studies have been mixed on the prognostic implications of HER2-low expression in breast cancer, and most evidence does not support any significant prognostic impact. In a large Korean cohort of early-stage patients, there was no significant difference in survival between HER2-low and HER2-0 breast cancers [23]. Another early-stage, Asian population study identified better prognosis in patients with HER2-low breast cancer in comparison with HER2-0, although the absolute difference was modest and driven predominately by IHC 1+ tumors, with no significant difference in survival for HER2 IHC 2+ compared with HER2-0 [24]. In a pooled analysis of patients from four neoadjuvant treatment trials, HER2-low was associated with improved disease-free survival (DFS) and overall survival (OS) compared with HER2-0. However, in subgroup analysis, survival differences were only seen in the HR-negative subgroup [8].The majority of early-stage studies have not demonstrated any differences in prognosis between HER2-low and HER2-0 breast cancer when considering HR-status [15, 25–29]. Other studies have shown differences in prognosis between HER2-low and HER2-0 for a certain subsets of patients, like HR positive breast cancers with high genomic risk [30] (Table 2).

For the advanced setting, a large cohort study of 15,054 patients observed a slight improvement in OS for HER2-low compared with HER2-0 breast cancer, particularly in the HR-negative subgroup [31]. Another cohort study of 1433 patients demonstrated improved OS for HER2-low versus HER2-0 but only among HR-positive patients [32]. Smaller cohort studies have not found significant differences in prognosis between HER2-low and HER2-0 advanced breast cancers [16, 33–35]. In our study of 749 patients with MBC, we also found no difference in prognosis between HER2-low and HER2-0 tumors; neither in the whole cohort nor stratified by HR status [5]. Table 2 summarizes prognostic findings in HER2-low breast cancers from the existing literature, including comparison of outcomes between HER2-low and HER2-0, and stratified analyses by HR-status, where available.

In summary, there has been no consistent prognostic impact or characteristic genomic features which distinguish HER2-low from HER2-0 breast cancers, particularly when accounting for HR status (Tables 1 and 2). This is unlike HER2-positive breast cancer, which has well described genomic features and prognostic implications that distinguish this subtype from HER2-negative breast cancers. If HER2-low were truly a unique biologic subtype, we would expect to see consistent findings supporting this across clinical studies. The only consistent finding across studies is that the clinical and prognostic features of HER2-low breast cancers depend predominantly on HR status. There are several limitations with the available data which are worth mentioning. Present studies comparing HER2-low and HER2-0 breast cancers are retrospective, and thus any conclusions about the prognostic implications of low HER2 expression are limited by bias inherent to retrospective analyses. Additionally, these studies lack central pathology review, and thus differences in methodologies and interobserver variability could result in misclassification of HER2-low or HER2-0. Lastly, we know that HER2 expression can change over time, with some HER2-0 tumors turning HER2-low and vice versa [29, 38]. This is fundamentally different than HER overexpression/amplification, which is linked to a definitive genomic aberration and relatively stable over time. The available studies do not consider temporal and spatial heterogeneity of HER2 expression and therefore the impact of this on prognosis remains unknown.

## DEFINING HER2-LOW STATUS

**Figure 1:**
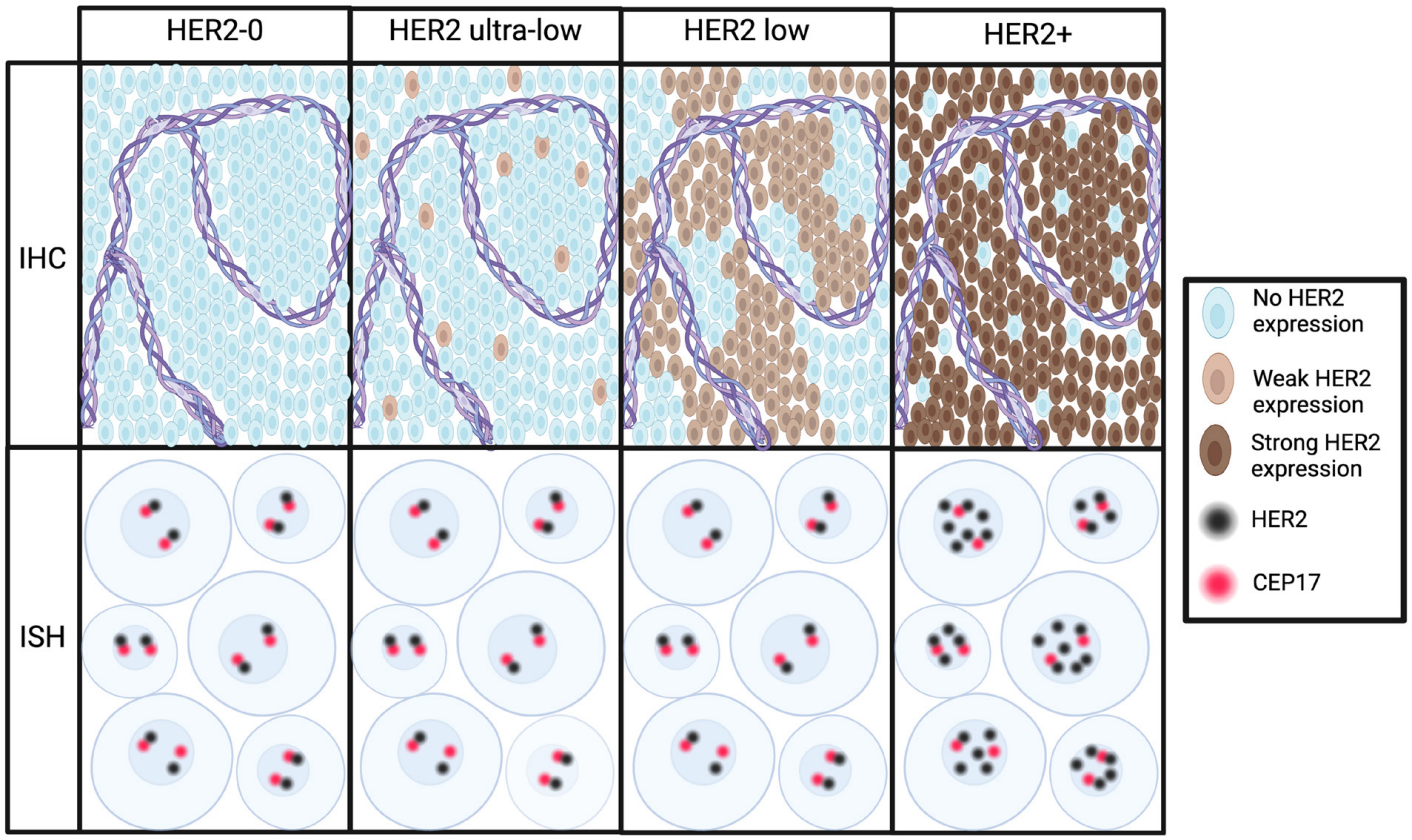
Immunohistochemistry (IHC) and in-situ hybridization (ISH) techniques for defining HER2 status. Increasing levels of HER2 expression in breast cells by IHC (top panel) correspond to an increased number and a deeper staining of HER2 on the cell surface. In HER2-0, ultra-low, and low, there is a single copy of HER2 on chromosome 17, which is marked by the CEP17 on ISH (bottom panel). In HER2-positive cells, extra copies of HER2 can be expressed.

The current definition of HER2-low is derived from ASCO/CAP guidelines for HER2 testing, which were developed to identify HER2-overexpressing tumors rather than accurately differentiating between HER2 scores in the low range [2] (Figure 1). There are several limitations with the current definition of HER2-low (and ultra-low) in breast cancer, including poor concordance in HER2 IHC scoring among pathologists at the 0/1+ level, spatial and temporal heterogeneity in HER2 low expressing tumors, and the fact that current IHC based tissue testing was never designed or validated to accurately differentiate between HER2 scores in the low range.

There is very poor concordance among pathologists in classifying tumors as either IHC score 1+ or 0 [39]. In a multi-institutional assessment of pathologist scoring HER2 using IHC, substantial discordance was observed between the intermediate categories (IHC 1+ and IHC 2+) with less than 1% and 3.6% agreement, respectively. Within the IHC 0 category, discordance was also substantial, with agreement of only 25% [40]. Lambein et al., found that central reassessment of breast cancers scored as IHC negative after local laboratory testing resulted in a shift of score IHC 0 to 1+ [41].

In DESTINY-Breast04, both fresh and archival biopsies were acceptable for enrollment. Prat et al., evaluated tissue submitted for central HER2 testing and included a total of 557 patients who were ultimately enrolled in DESTINY-Breast04. While most of these samples were from metastatic sites, 35% of patients submitted primary tumor samples. Most samples were biopsy specimens from archival tissue (88%) rather than fresh biopsies. Efficacy of T-DXd was consistent across all tumor sample characteristics [42]. Real-world efficacy of T-DXd was evaluated in the RELIEVE study, which included 191 patients treated with T-DXd at two academic centers between 2017 and 2022. Investigators noted that HER2-low expression is an unstable biomarker, and often changes during treatment. Patients treated with T-DXd whose HER2 expression changed from HER2-low to HER2-0 from the primary to metastatic setting had a median time-to-next treatment (TTNT) of only 3.0 months, whereas it was 5.6 months when switching from HER2-0 to HER2-low, and 9.4 months for stable HER2-low expression (*P* = 0.006). Among 25 patients who demonstrated stable HER2-low expression from initial metastatic biopsy to pre-T-DXd treatment biopsy, the TTNT was 8.6 months [43]. At this time, we favor the definition of HER2-low be applied to tumors found to have low HER2 expression at any time point. However, more data are needed to refine the definition of HER2-low according to the temporal evolution of the biomarker.

The current definition of HER2 IHC 0 allows for a faint, barely perceptible expression in less than 10% of tumor cells, which could be classified as HER2 “ultra-low” (Figure 1). The phase 2 DAISY trial revealed an objective response rate (ORR) of 30% among 37 patients with HER2 IHC 0 MBC treated with T-DXd [44]. Initial report from the ongoing DESTINY-Breast06 phase 3 trial, revealed significant response to T-DXd in the HR-positive HER2 “ultra-low” population [45], thus paving the way for future expansion of drug eligibility to patients with minimal expression of HER2.

## DEFINING HER2-LOW STATUS: FUTURE PERSPECTIVES

Alternative methodologies have been proposed to quantitatively detect HER2 protein expression in tumor tissue, including HERmark^™^ and other immunofluorescence-based assays [46–51], real-time quantitative polymerase chain reaction (RT-PCR) [52–54], targeted mass spectrometry [55] and digital pathology [56–61].

The HERmark^™^ breast cancer assay was developed to precisely quantify HER2 protein expression using a dual-antibody, proximity-based, immunofluorescence approach and has been shown to have greater sensitivity and specificity than IHC, providing a continuum of HER2 protein expression values over approximately 1,000-fold dynamic range in human breast cancers and cell lines [46]. It has been shown to correlate with HER2 protein expression and survival in clinical studies [47]. The Enhanced Sensitivity HERmark^™^ assay (ESHA) was developed to quantify low HER2 protein expression, optimizing the assay to low-expressing condition using low HER2 expressing cancer cell lines, which extended the lower end of the dynamic range of the HERmark^™^ assay and decreased assay variation [48].

Multiple-reaction monitoring mass spectrometry (MRM-MS) has been shown to correlate well with IHC/ISH and with clinical response to HER2-targeted therapies [62, 63]. Kennedy et al., used immunoaffinity-enrichment coupled with MRM-MS (immune-MRM-MS) to quantify HER2 protein expression in breast biopsies. The authors found excellent agreement between immune-MRM-MS measurements and HER2 status by IHC/ISH, particularly when measurements were normalized by glyceraldehyde-3-phosphate-dehydrogenase (GAPDH) (a surrogate for tumor cellularity). They were able to detect HER2 above the lower limit of quantification in all study samples, including HER2 negative (IHC 0) and HER2-low tumors. Among the HER2-low and HER2 negative tumors, there was high correlation with MRM and both mRNA expression and ERBB2 copy number [55]. The findings showed this method enables precise, relative quantification of HER2 in HER2-low and HER2-negative tumors.

Other methods to improve HER2 assessment include machine learning approaches. Artificial intelligence (AI) computer algorithms can analyze images, quantify HER2 membrane staining, and generate reliable and reproducible results [60, 61]. Palm et al*.*, evaluated the performance of an AI-assisted workflow to determine HER2 status in primary and metastatic breast cancers, according to ASCO/CAP guidelines. This study revealed moderate agreement between pathologists and AI on IHC scores (Cohen’s k 0.59), with most discrepancies occurring between IHC 0 and IHC 1+ [59]. Sode et al., compared HER2 reported by digital image analysis (DIA) (device-only) or assisted reading (AR) (pathologist is informed of the DIA result) at a single-institution using 761 breast tumors IHC stained for HER2. Moderate agreement was seen between DIA and AR (Cohen’s k 0.66). Discordant cases showed heterogeneous and aberrant staining, representing a pitfall in the evaluation of HER2-low breast cancer. Of note, pathologists more commonly re-assigned a lower HER2 score (IHC 0 from IHC 1+) compared to DIA in the HER2-low subgroup [56]. Another multi-institutional study showed improved accuracy of HER2 IHC 0 and 1+ tumor interpretation when pathologists were assisted by AI compared to not being assisted (0.93 vs. 0.8). Importantly, accuracy significantly improved even in the presence of heterogeneity (0.89 vs. 0.68) [64]. Given increased heterogeneity in HER2 IHC 1+ and IHC 2+ compared to IHC 3+ tumors, this methodology could assist pathologists in a more precise HER2-low evaluation.

At present, there is no simple or efficient method to follow the changes in HER2 over time, as repeat invasive biopsies are unacceptable to patients and fail to overcome spatial and clonal heterogeneity. Prior studies have shown that molecular characterization of circulating tumor cells (CTCs) can be used to evaluate HER2 expression, among other biomarkers [65]. HER2-positive CTCs can be detected in patients with HER2-negative primary tumors [65–67] and have been associated with response to HER2 targeted therapies [68–71]. HER2 expression levels in CTCs may change as breast cancer progresses [72]. Initial work in CTCs focused on HER2 overexpression as categorized by *ERBB2* gene amplification or high IHC staining intensity (IHC 3+) [73]. However, in recent years, identifying CTCs with lower HER2 expression has become a focus. In a retrospective cohort study of patients with advanced breast cancer, the presence of HER2-low CTCs (IHC 1+) was correlated with worse prognosis and more aggressive metastatic behavior [74, 75]. Quantitative HER2 analysis methods are now in development to evaluate low levels of HER2 expression in individual circulating tumor cells (CTCs) [76].

## CLINICAL IMPLICATIONS OF HER2-LOW

HER2-low became relevant from a clinical perspective after the publication of results from the DESTINY-Breast04 trial. In this trial, patients with MBC and HER2 IHC 1+ or 2+ with negative ISH (“HER2-low”) were randomized to receive either T-DXd (HER2-directed ADC) or chemotherapy of physician’s choice (TPC) [1]. Compared with TPC, T-DXd resulted in significantly longer PFS and OS. The median PFS was 9.9 months with T-DXd versus 5.1 months for TPC (HR 0.50, 95% CI 0.40–0.63, *p* < 0.001). Median OS was 23.4 months with T-DXd versus 16.8 months for TPC (HR 0.64, 95% CI 0.49–0.84, *p* < 0.001). Benefit from T-DXd over TPC was observed in the HR-positive patients (*N* = 494), with median PFS 10.1 versus 5.4 months (HR 0.51, *p* < 0.001) and median OS 23.9 versus 17.5 months (HR 0.64, *p* = 0.003). In an exploratory analysis limited to TNBC (*N* = 58), benefit was also demonstrated with median PFS 8.5 versus 2.9 months (HR 0.46) and median OS 18.2 versus 8.3 months (HR 0.48) for T-DXd compared with TPC [1]. Drug-related interstitial lung disease (ILD) or pneumonitis occurred in 12% of patients receiving T-DXd, with 0.8% fatal events representing a key side effect of concern with this compound [1].

Sacituzumab govitecan (SG) is another ADC, targeting anti-trophoblast cell surface antigen 2 (TROP2), that was approved for metastatic TNBC based on the results of the phase 3 ASCENT randomized clinical trial. Among 486 patients, those assigned to SG derived an improvement in OS compared to TPC (12.1 versus 6.8 months; HR 0.48, 95% CI 0.38–0.59) [77]. SG has also been approved for pretreated metastatic HR-positive breast cancer, based on the results of the phase 3 TROPICS2 clinical trial. Among 543 patients, those assigned to SG derived an improvement in OS compared to TPC (14.4 versus 11.2 months; HR 0.79, 95% CI 0.65–0.96) [78, 79]. In subgroup analyses of the ASCENT and TROPICS clinical trials, consistent benefit with Sacituzumab govitecan was seen for HER2-low and HER2-0 breast cancers [80, 81]. Of note, patients in both ASCENT and TROPICS2 were more heavily pretreated than those enrolled in DESTINY-Breast04. SG and T-DXd both carry a topoisomerase I inhibitor payload, which raises concern about cross-resistance with their sequential use [82]. There are no prospective comparison studies available for the two agents, leaving the choice of which ADC to administer up to the treating physician after careful appraisal of the available data and shared decision-making.

**Figure 2:**
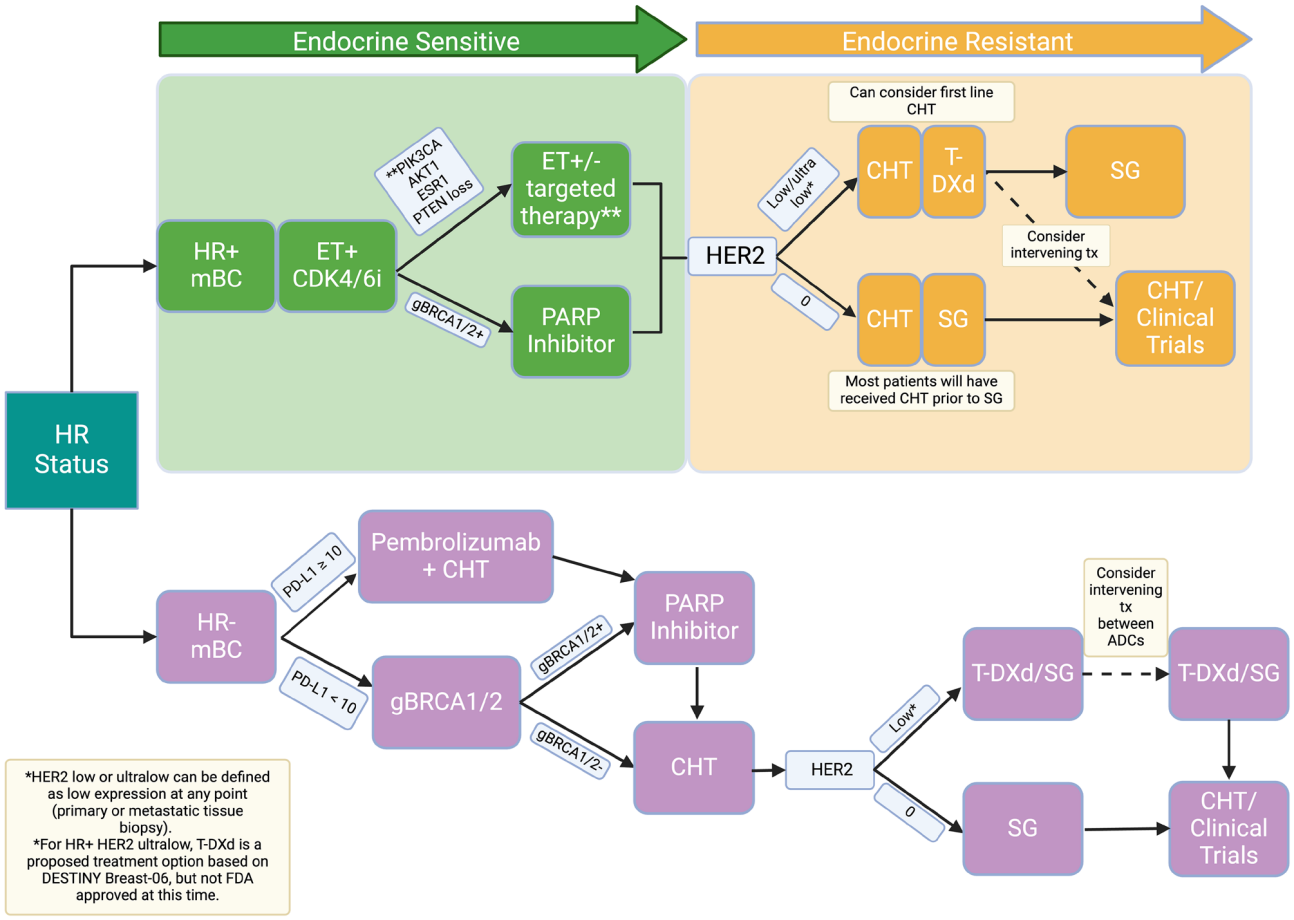
Proposed treatment algorithm by HR status. Our proposed treatment algorithm reflects a potential treatment option for HR+, HER2 ultralow metastatic breast cancer based on the results of DESTINY Breast-06. HR: hormone receptor; mBC: metastatic breast cancer; ET: endocrine therapy; CHT: chemotherapy; T-DXd: trastuzumab deruxtecan; SG: sacituzumab govitecan; ADCs: antibody drug conjugate; tx: treatment.

Results of the phase 3 trial DESTINY-Breast06 were reported at ASCO 2024. This study evaluated T-DXd in HER2-low and HER2-ultralow HR+ breast cancer with no prior exposure to chemotherapy. T-DXd significantly improved PFS compared to TPC (majority capecitabine) in HER2-low (HR 0.62, *p* < 0.0001, median 13.2 vs. 8.1 months) and HER2-ultralow (HR 0.78, median 13.2 vs. 8.2 months) breast cancers with an objective response rate (ORR) to T-DXd of 56.5% and 61.8%, respectively [45]. OS data was still immature at this interim analysis, with median follow-up of 18.6 months. Of note, there was an increase in grade ≥3 adverse events with T-DXd compared to TPC (40.6% vs. 31.4%); including higher incidence of ILD (11.3% vs. 0.2%) and 3 ILD-related deaths [45]. These data indicate that T-DXd could become the preferred first-line treatment after endocrine-resistance among patients with HR-positive and HER2-low breast cancer. Perhaps the most significant implications will be the expansion of T-DXd indication to include those patients with ultra-low HER2 expression. Considering these results, we have proposed the below treatment algorithm for patients with HER2 negative MBC (Figure 2).

## CLINICAL IMPLICATIONS OF HER2-LOW: FUTURE PERSPECTIVES

Results from DESTINY-Breast06 among the HER2 ultra-low population raise the question as to whether HER2 expression is needed at all for T-DXd efficacy in breast cancer. SG, for example, has been studied in cancers known to overexpress TROP2. However, no difference in efficacy has been demonstrated between TROP2-high or TROP2-low breast cancers [77]. Several studies have suggested that HER2-expression is indeed an important indicator for treatment efficacy with T-DXd. In the RELIEVE study, changes in HER2-expression between primary and metastatic biopsies were associated with response (assessed by TTNT) to T-DXd. Those with stable HER2 low expression appeared to experience the greatest benefit, with 9.4 months TTNT [43].

## ADC SEQUENCING

Several studies have evaluated sequencing ADCs in advanced breast cancers and specifically in HER2-low breast cancers [83–87]. In general, efficacy appears to be greatest with the first ADC. There is evidence of cross-resistance when patients are treated with a second ADC, especially as available ADCs utilize the same topoisomerase inhibitor payload. One of the proposed mechanisms of cross-resistance is acquired mutations in TOP1 gene [83]. There is no consensus on whether adding an intervening line of treatment can overcome cross-resistance. Prospective randomized trials to address sequential ADC administration with a switch in targets are planned. The TRADE-DXd trial will include patients with HER2-low breast cancer who will receive treatment with ADC1 (T-DXd; HER2 target or Dato-DXd; TROP2 target) with crossover to the opposite ADC (ADC2) at progression. This will answer questions about optimal sequencing and mechanisms of resistance when ADCs with the same payload, but different targets are utilized.

## NOVEL ADCs AND COMBINATIONS

Additional efforts to broaden the applicability of anti-HER2 ADCs in HER2-low breast cancer are currently underway. This includes combinations involving endocrine therapy, chemotherapy, and immunotherapy with T-DXd [88] as well as novel ADCs with different linker technologies and cytotoxic payloads [89–93]. Combining HER2-directed ADCs, such as T-DXd, with HER2 tyrosine kinase inhibitors, such as neratinib, have shown synergistic treatment effect in preclinical models due to increased internalization of the ADC payload [94, 95]. This combination is currently being evaluated in a phase I study for patients with advanced solid tumors harboring alterations in HER2 [96]. DESTINY-Breast07 is a multi-cohort phase 1b/2 study combining T-DXd with other agents in HER2-positive breast cancers, and results from the T-DXd + pertuzumab dose-expansion arm were presented at ASCO 2024. ORR for the combination was 82% among 50 patients and PFS at 12 months was 77.3% [97]. DESTINY-Breast08 is multi-cohort phase 1b study evaluating T-DXd in combination with other agents, including chemotherapy (capecitabine, paclitaxel plus durvalumab), endocrine therapy (anastrozole, fulvestrant) and targeted-therapy (capivasertib) [88]. Preliminary results indicate that T-DXd can be safely used in combination with anastrozole and fulvestrant [98]. Evaluation of T-DXd in combination with immunotherapy is also underway in several phase 1 trials with nivolumab [99], durvalumab [100] and pembrolizumab [101] with preliminary signals of efficacy.

In combined analysis of C001CANCER and C003cANCER phase 1 studies utilizing RC48, anti-HER2 ADC with monomethyl auristatin E cytotoxic payload, an ORR of 39.6% and median PFS of 5.7 months was demonstrated in the HER2-low cohort [89, 90]. SYD985, anti-HER2 ADC with duocarmycin analog cytotoxic payload, demonstrated partial response in 28% of patients with HER2-low, HR-positive breast cancer and 40% of patients with HER2-low, HR-negative breast cancer [91]. In a phase 1 study, A166, anti-HER2 ADC with microtubule inhibitor cytotoxic payload, demonstrated disease control rate of 75% among the 4 evaluable patients with HER2-low breast cancer [93].

## CONCLUSIONS

Breast cancer, which has been historically classified as HER2-positive versus HER2-negative, is currently facing a paradigm shift in both the definition of HER2 status and in the existing treatment algorithms. Despite evidence from existing literature that HER2-low breast cancer does not represent a distinct biologic and prognostic subtype, the introduction of HER2-low expression as a therapeutic target has expanded patient eligibility for a potent class of anti-HER2 drugs, HER2-directed ADCs, with potential for significant efficacy.

There are several challenges with the current IHC-based definition of HER2-low, including poor concordance between pathologists and heterogeneity in HER2 expression. The current methodology was designed to detect HER2 overexpression and was never intended to differentiate HER2 scores in the low range. HER2 testing needs to be improved to more quantitatively identify levels of HER2 expression. Standardization and validation of new HER2 testing methods will improve concordance with HER2 scoring in the low range and expand the spectrum of actionable HER2 expression. CTC-based testing has the potential to address spatial and temporal heterogeneity in HER2 expression. Understanding the impact of this heterogeneity on efficacy of HER2-directed ADCs is critical to refine patient selection in clinical practice.

Despite significant efficacy with T-DXd demonstrated in DESTINY-Breast04, many questions remain about how to best position this compound within existing treatment paradigms. Next generation ADCs, including compounds directed against HER2 with improved linker technologies and different cytotoxic payloads, are currently in development. In the absence of head-to-head comparisons and prospectively designed sequencing studies, the choice of which treatments to use will rely on a critical appraisal of available data, understanding of possible cross-resistance with sequential use of ADCs, and shared decision-making.
